# Epidemiological changes in *Toxoplasma* infection: a 7-year longitudinal study in pregnant women in Lyon, France, 2017–2023

**DOI:** 10.1051/parasite/2025023

**Published:** 2025-05-21

**Authors:** Alvaro Roy, Laurent Gaucher, Damien Dupont, Jean Menotti, Anthony Atallah, Benoit de la Fournière, Mona Massoud, Bruno Lina, Pauline Tirard-Collet, Martine Wallon

**Affiliations:** 1 Department of Virology, Institute for Infectious Agents, Hospices Civils de Lyon 103 Grande Rue de la Croix-Rousse 69004 Lyon France; 2 National Centre for Epidemiology, Instituto de Salud Carlos III Avenida de Monforte de Lemos 5 28029 Madrid Spain; 3 Geneva School of Health Sciences, HES-SO University of Applied Sciences and Arts Av. de Champel 47 1206 Geneva Switzerland; 4 Department of Maternal Fetal Medicine, University Hospital Femme Mère-Enfant, Hospices Civils de Lyon 59 Boulevard Pinel 69500 Bron France; 5 Research on Healthcare Performance (RESHAPE), INSERM U1290, Claude Bernard University Lyon 1 8 Avenue Rockefeller 69373 Lyon France; 6 Department of Parasitology and Medical Mycology, Institute for Infectious Agents, Hospices Civils de Lyon 103 Grande Rue de la Croix-Rousse 69004 Lyon France; 7 WAKING Team, Lyon Neuroscience Research Center, Claude Bernard University Lyon 1 95 Boulevard Pinel 69675 Bron France; 8 CarMeN Lab, team IRIS INSERM 1060, Claude Bernard University Lyon 1 59 Boulevard Pinel 69500 Bron France; 9 Department of Gynecology and Obstetrics, Hôpital de la Croix-Rousse, Hospices Civils de Lyon 103 Grande Rue de la Croix-Rousse 69004 Lyon France; 10 LabTau, Inserm U1032, Laboratory of Therapeutic Applications of Ultrasound, Claude Bernard University Lyon 1 151 Cours Albert Thomas 69424 Lyon France; 11 Fetal Medicine Unit, Hôpital Lyon Sud, Hospices Civils de Lyon 165 Chemin du Grand Revoyet 69310 Pierre-Bénite France; 12 FLUID Team, Lyon Neurosciences Research Center, INSERM U1028, CNRS UMR5292, Claude Bernard University Lyon 1 95 Boulevard Pinel 69675 Bron France

**Keywords:** Toxoplasmosis, *Toxoplasma gondii*, Pregnancy, Seroprevalence, Incidence, France

## Abstract

The epidemiology of *Toxoplasma* infection is known to vary geographically, but is also likely to vary over time, under the influence of many contributing factors. Monitoring is particularly useful in the context of preventing congenital toxoplasmosis. We took advantage of the French prenatal prevention programme to retrospectively assess changes between 2017 and 2023 in seroprevalence and incidence rates of *Toxoplasma* infection in pregnant women and the incidence of congenital infections. We conducted a longitudinal retrospective study including all pregnancies with known *Toxoplasma* status followed up at Lyon’s public maternity hospitals between 2017 and 2023 (71,922 pregnancies). We used a multivariable logistic regression model to identify factors (age-group, WHO region of origin, population density of the area of residence and parity) associated with seropositivity. The seroprevalence of toxoplasmosis decreased consistently from 26.4% in 2017 to 22.1% in 2023 (*p* = 0.003), while maternal infection incidence remained stable at 1.3/1,000 pregnancies at risk. Notably, the seroprevalence showed a linear increase with age from 18.9% in women aged 25–29 years to 38.0% in women aged ≥40 years (*p* < 0.001). The seroprevalence was lower in pregnant women living in rural areas [adjusted seroprevalence ratio (aPR) = 0.87, 95% CI: 0.82–0.92] and higher in multiparous women (aPR = 1.08, 95% CI: 1.04–1.12). This study confirms the ongoing decline in toxoplasmosis seroprevalence while seroconversions remained stable, indicating a need for more tests in seronegative women in the future. These findings highlight the need for ongoing monitoring and refinement of congenital toxoplasmosis prevention strategies in high-income countries.

## Abstract (fr)

Il est connu que l’épidémiologie de l’infection à *Toxoplasma* varie selon les zones géographiques, mais elle est également susceptible de varier au fil du temps, sous l’influence de nombreux facteurs contributifs. La surveillance est particulièrement utile dans le cadre de la prévention de la toxoplasmose congénitale. Nous avons exploité le programme français de prévention prénatale pour évaluer rétrospectivement l’évolution entre 2017 et 2023 de la séroprévalence et des taux d’incidence de l’infection à *Toxoplasma* chez les femmes enceintes, ainsi que de l’incidence des infections congénitales. Nous avons mené une étude rétrospective longitudinale incluant toutes les grossesses dont le statut toxoplasmique était connu, suivies dans les maternités publiques de Lyon entre 2017 et 2023 (71 922 grossesses). Nous avons utilisé un modèle de régression logistique multivariée pour identifier les facteurs (tranche d’âge, région d’origine OMS, densité de population du lieu de résidence et parité) associés à la séropositivité. La séroprévalence de la toxoplasmose a diminué de façon constante, passant de 26,4 % en 2017 à 22,1 % en 2023 (*p* = 0,003), tandis que l’incidence de l’infection maternelle est restée stable à 1,3/1 000 grossesses à risque. Il est à noter que la séroprévalence a montré une augmentation linéaire avec l’âge, passant de 18,9 % chez les femmes âgées de 25 à 29 ans à 38,0 % chez les femmes âgées de plus de 40 ans (*p* < 0,001). La séroprévalence était plus faible chez les femmes enceintes vivant en zone rurale [rapport de séroprévalence ajusté (aPR) = 0,87, IC à 95 % : 0,82-0,92] et plus élevée chez les femmes multipares (aPR = 1,08, IC à 95 % : 1,04-1,12). Cette étude confirme la baisse continue de la séroprévalence de la toxoplasmose, tandis que les séroconversions sont restées stables, ce qui indique la nécessité de réaliser davantage de tests chez les femmes séronégatives à l’avenir. Ces résultats soulignent la nécessité d’une surveillance continue et d’un perfectionnement des stratégies de prévention de la toxoplasmose congénitale dans les pays à revenu élevé.

## Introduction

Toxoplasmosis is caused by the ubiquitous protozoan parasite *Toxoplasma gondii*, which is present in both the environment in the form of oocysts eliminated via the faeces of felids, and capable of resisting in soil, water and food, and in the form of cysts present in the undercooked meat of farmed or wild animals [24]. Infection is usually subclinical or benign in the immunocompetent host, but can have severe consequences in immunocompromised patients or in subjects contaminated *in utero* in the context of a maternal infection contracted during pregnancy or shortly before. Congenital toxoplasmosis is associated with foetal loss, neonatal death and neurological and ocular complications in the newborn [14]. However, effective treatment is available and can mitigate the risk of maternal-fetal transmission in case of maternal seroconversion [9].

Prevention strategies include health education, retesting susceptible pregnant women for early diagnosis and treatment of infection, and neonatal screening. Seroprevalence is an essential parameter for targeting these preventive measures and assessing their cost-effectiveness, as it varies considerably from region to region and within regions throughout the world, depending on climate, altitude, livestock production and dietary habits [4]. International data show a decrease in the seroprevalence of *Toxoplasma* in high-income countries [15]. This decline in prevalence may have several causes related to reduced exposure to different parasite stages, including health education, urbanisation, fewer contaminated cats, modernisation of farming and agri-food production conditions, and lifestyle and hygiene changes [6, 15]. However, longitudinal data remain limited, even in countries with prenatal screening such as France, which relies only on six national cross-sectional surveys since 1995 [10].

There is also sparse recent data on the incidence of maternal and congenital infections in this context of declining prevalence, which may be counterintuitive since it also depends on parasite genotype, population demographic structure and local epidemiology [15]. In France, the proportion of seroconversions among pregnant women remained at 0.2% in 2016 and 2021 according to the National Perinatal Survey [10]. The incidence of congenital toxoplasmosis cases slightly decreased from 3.3 cases per 10,000 births in 2007, to 2.1 cases in 2017 and to 1.9 cases in 2022 in France [22].

To answer these questions, we wanted to draw on data routinely collected as part of the French National Prevention Programme for Congenital Toxoplasmosis over a period of seven consecutive years at the second largest university hospital in France (serving a population of more than 1.4 million and about 10,000 births per year to women from urban, semi-rural and rural areas). Herein, this study aims to assess the longitudinal changes in seroprevalence in pregnant women in Lyon, France, from 2017 to 2023. Secondary objectives were to assess the incidence of maternal and congenital infections in the same population.

## Methods

### Ethics statement

This non-interventional study used data collected as part of routine care. No additional data collection was necessary. Patient information was anonymised and coded prior to inclusion in the database and analysis, as well as institution and service. All documents and databases were deposited only on internal servers and opened under password security. According to the French Health Public Law (CSP Art L1121-1.1), such protocols do not require approval by an ethics committee and are exempt from the otherwise mandatory informed consent requirements. Nevertheless, the study protocol has been submitted and approved by the *ad hoc* ethics committee of the HCL (CSE-HCL – IRB 00013204; approval number 22-5110, 02/12/2023).

### French National Prevention Programme

The study was performed in the context of the French National Prevention Programme for Congenital Toxoplasmosis, which since 1985 provides for women at risk to be identified and informed on how to avoid toxoplasmosis during their first trimester of pregnancy, and since 1992 to undergo monthly serological monitoring until delivery. Serological tests for the simultaneous detection of anti-toxoplasmic IgG and IgM are fully reimbursed, with no advance payment required. They are carried out in private laboratories, particularly at the start of pregnancy, or in private or public obstetrics and gynaecology departments for the following prenatal tests and those carried out at delivery. Patients can be referred to expert centres for counselling and prenatal diagnosis procedures including foetal ultrasound and amniocentesis.

The following tests were available for establishing IgG seropositivity, diagnosing acute maternal infections and estimating the gestational age of maternal infection and diagnosing congenital toxoplasmosis, according to patient profile: Architect^®^ Toxo IgG and IgM (Abbott Laboratories, Abbott Park, IL, USA), VIDAS^®^ Toxo IgG and avidity (BioMérieux, Lyon, France), Platelia™ IgM and IgA (Bio-Rad, Marne-la-Coquette, France), Toxo II Western Blot IgG (LDBio, Lyon, France) and Toxo comparative Western Blot for IgG and IgM (LDBio, Lyon, France). All tests were performed and their results interpreted according to manufacturer’s recommendations.

### Case definition

A woman was considered seronegative if she had documented absence of IgG antibodies and seropositive if she had documented presence of IgG antibodies or documented seroconversion during pregnancy. Maternal infection was defined as a change from IgG negative to IgG positive or a significant increase in IgG by ARCHITECT^®^ Toxo IgG and VIDAS^®^ Toxo IgG tests, in the presence of elevated IgM titres. Infections estimated prior to conception were not included. Congenital toxoplasmosis was confirmed in cases of positive PCR in amniotic fluid, positive results for ultrasensitive IgM (Platelia™ Toxo IgG) or IgA (Platelia™ TOXO IgA TMB, Bio-Rad, France) tests at three days of age or persistently positive IgG at one year of age.

### Data collection

Our study was longitudinal and included all women giving birth at Lyon’s university hospitals between 1 January 2017 and 31 December 2023. As early miscarriages (before 14 weeks of amenorrhoea) and voluntary terminations of pregnancy for reasons of convenience were the responsibility of the gynaecology department and not the maternity department, they were not included in the study. Voluntary terminations of pregnancy for medical reasons were included.

### Date processing and validation

Age in years was calculated from the mother’s date of birth and her date of delivery. The country of birth and postcodes were obtained from the patients’ administrative records. Countries of birth were grouped into WHO regions: African Region (AFR), Region of the Americas (AMR), South-East Asian Region (SEAR), European Region (EUR), Eastern Mediterranean Region (EMR), and Western Pacific Region (WPR). The postcode of residence was used to estimate the population density of the place of residence based on 7-level municipal density grid of the *Institut National de la statistique et des études économiques* (INSEE) [13]. Levels were grouped into urban areas (level 1: large city; level 2: medium-sized city; level 3: small city; and level 4: city belt) and rural areas (level 5: rural towns; level 6: rural with scattered settlements; and level 7: rural with widely scattered settlements).

### Statistical analysis

We calculated the seroprevalence and seroconversions (maternal infections) per year by sociodemographic variables throughout the study period. Annual incidence rates for maternal and congenital infections were calculated based on the year of delivery. Temporal trends in seroprevalence rates across age groups were analysed using Mann-Kendall test. Univariate and multivariable logistic regression was used to estimate annual seroprevalence and assess associations with age, parity, WHO region of birth, and urban or rural residence. Analyses were conducted using R software, version 4.1.3 (R Foundation for Statistical Computing, Vienna, Austria).

## Results

### Seroprevalence

Information on toxoplasmosis serological status was available for 71,922 women between 2017 and 2023 (*i.e.* 97% of the women registered in the database) residing both in urban and rural areas. Among women with a known serological status, the median age [IQR] was 32 years [29–35], 45.5% were first pregnancies and 3.1% were pregnancies with multiple births. The seroprevalence of toxoplasmosis decreased consistently from 26.5% in 2017 to 22.1% in 2023 (*p* for trend = 0.003; Table 1).

**Table 1:** Seroprevalence in pregnant women with known *Toxoplasma* status who gave birth in Lyon’s public hospitals between 2017 and 2023.

*Toxoplasma* status*	2017		2018		2019		2020		2021		2022			2023			Overall	
	*n*	%	*n*	%	*n*	%	*n*	%	*n*	%	*n*	%	*n*		%	*n*		%
Negative	7,134	73.5	7,870	74.6	8,056	74.8	7,912	75.6	7,941	76.0	7,882	77.7	7,647		77.8	54,442		75.7
Positive	2,563	26.4	2,668	25.3	2,714	25.2	2,540	24.3	2,501	23.9	2,251	22.2	2,174		22.1	17,411		24.2
Seroconversion	9	0.09	10	0.09	6	0.06	11	0.11	13	0.12	11	0.11	9		0.09	69		0.10
Total	9,706		10,548		10,776		10,463		10,455		10,144		9,830			71,922		

*Pregnant women with missing values (*n*, %): 2017 (397, 3.9%), 2018 (353, 3.2%), 2019 (330, 3.0%), 2020 (298, 2.8%), 2021 (269, 2.5%), 2022 (266, 2.6%), 2023 (215, 2.1%).

**Figure 1:**
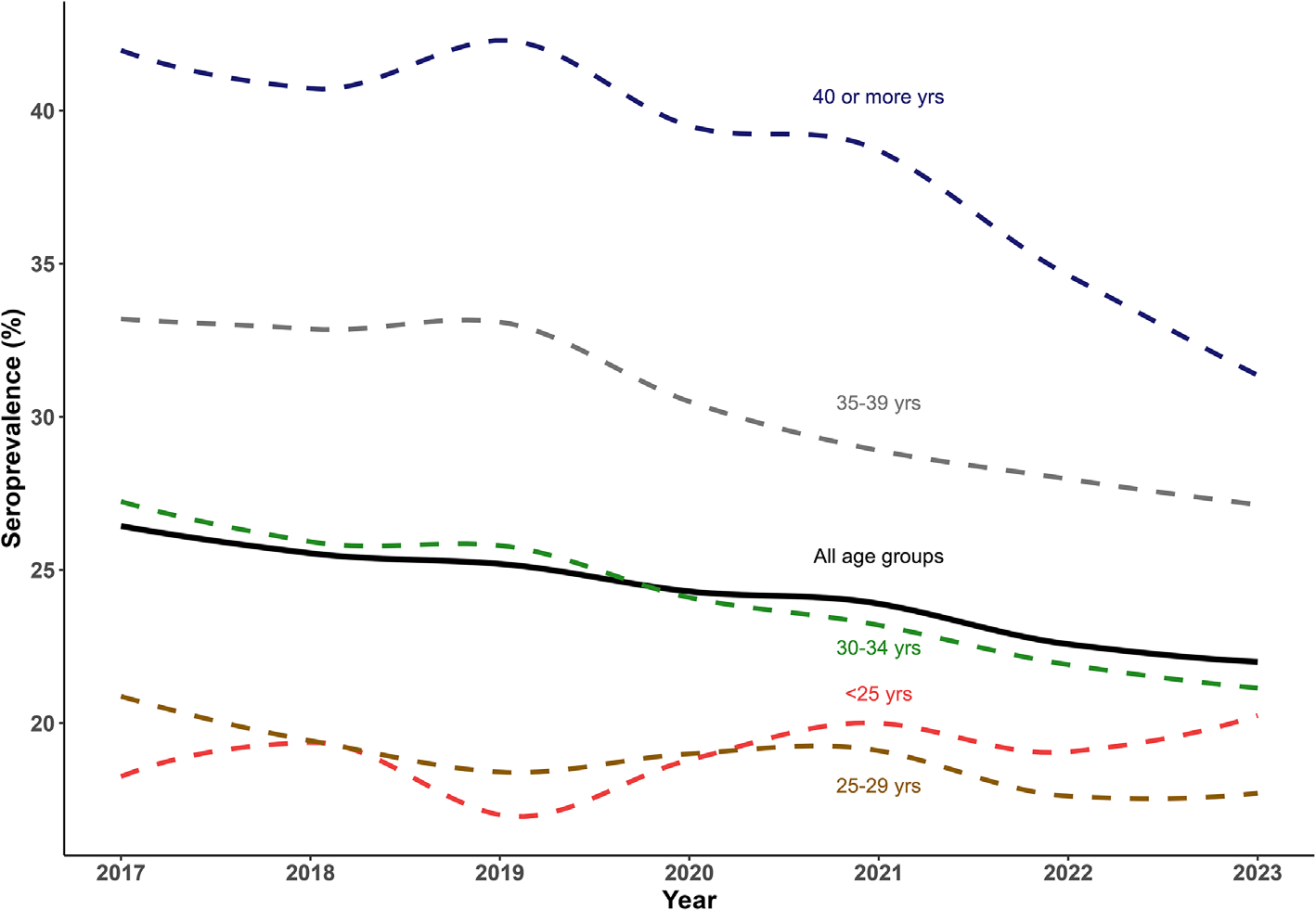
Seroprevalence (%) of toxoplasmosis stratified by age group in pregnant women with known *Toxoplasma* status who gave birth in Lyon’s public hospitals between 2017 and 2023.

The decreasing trend was observed specifically in the age groups of 30–34, 35–39 and ≥40 years old (*p* < 0.05; Fig. 1), but not in age groups <30 years, where no trend was detected. Herein, the seroprevalence increased with age, with the odds of being seropositive almost 2-fold higher in the 40 or more years old group as in the 30–34 age group (*p* < 0.001; Table 2). In the multivariable analysis, the seroprevalence was lower in pregnant women living in rural areas [urban = 24.9% and rural = 19.8%; adjusted seroprevalence ratio (aPR) = 0.87, 95% CI: 0.82–0.92] and higher in multiparous women (aPR = 1.08, 95% CI: 1.04–1.12). In addition, pregnant women born in the WHO AFR (39.9%), EMR (33.2%) and EUR (24.9%) regions showed a higher adjusted seroprevalence ratio than in France (20.6%) (*p* < 0.001). In contrast, women born in the WPR showed the lowest seroprevalence, at 9.1% (Table 2).

**Table 2:** *Toxoplasma* seroprevalence in pregnant women and demographic characteristics associated with seropositivity, Lyon, 2017–2023 (*n* = 71,922).

Variables	*n*	%	Seroprev., %	Crude PR	95% CI	*p*-value	aPR	95% CI	*p*-value
Age (years)									
<25	6,530	9.1	19.1	0.74	0.69–0.79	**<0.001**	0.66	0.62–0.71	**<0.001**
25–29	19,231	26.7	18.9	0.73	0.70–0.77	**<0.001**	0.71	0.68–0.74	**<0.001**
30–34	27,228	37.9	24.1	Ref.	Ref.	Ref.	Ref.	Ref.	Ref.
35–39	15,149	21.1	30.4	1.37	1.31–1.44	**<0.001**	1.33	1.27–1.39	**<0.001**
≥40	3,784	5.3	38.0	1.93	1.79–2.07	**<0.001**	1.78	1.65–1.91	**<0.001**
Total	71,922	100.0	NA	NA	NA	NA	NA	NA	NA
Place of residence									
Urban area	63,244	88.0	24.9	Ref.	Ref.	Ref.	Ref.	Ref.	Ref.
Rural area	8,625	12.0	19.8	0.75	0.71–0.79	**<0.001**	0.87	0.82–0.92	**<0.001**
Total	71,869	100.0	NA	NA	NA	NA	NA	NA	NA
Parity									
First pregnancy	32,751	45.5	21.2	Ref.	Ref.	Ref.	Ref.	Ref.	Ref.
≥2	39,162	54.5	26.9	1.37	1.33–1.42	**<0.001**	1.08	1.04–1.12	**<0.001**
Total	71,913	100.0	NA	NA	NA	NA	NA	NA	NA
Country/WHO Region of birth									
France	51,514	72.5	20.6	Ref.	Ref.	Ref.	Ref.	Ref.	Ref.
African Region (AFR)	10,282	14.5	39.9	2.55	2.44–2.67	**<0.001**	2.54	2.43–2.66	**<0.001**
Region of the Americas (AMR)	659	0.9	21.9	1.08	0.89–1.29	0.4	0.98	0.81–1.18	0.859
Eastern Mediterranean Region (EMR)	3,844	5.4	33.2	1.91	1.78–2.05	**<0.001**	1.85	1.72–1.99	**<0.001**
European Region (EUR)	3,913	5.5	24.9	1.27	1.18–1.37	**<0.001**	1.30	1.21–1.41	**<0.001**
South-East Asian Region (SEAR)	201	0.3	19.4	0.93	0.64–1.30	0.7	0.89	0.616–1.25	0.511
Western Pacific Region (WPR)	616	0.9	9.1	0.38	0.29–0.50	**<0.001**	0.35	0.26–0.46	**<0.001**
Total	71,029	100.0	NA	NA	NA	NA	NA	NA	NA

Seroprev.: seroprevalence; Crude PR: crude prevalence ratio; aPR: adjusted prevalence ratio; Bold *p*-values indicate statistical significance

### Incidence of maternal infections and congenital toxoplasmosis

Sixty-nine gestational infections were observed during 2017–2023, which resulted in a cumulative incidence of 1.3 cases [95% CI: 0.99–1.61] per 1,000 pregnancies at risk. Annual incidence rates remained constant at 0.1% or below throughout the period (Table 1). There were no terminations of pregnancy. Maternal infections led to congenital toxoplasmosis in eight cases, resulting in one foetal death, and in the detection at birth of intra-cranial calcifications in one of the seven live births. All children were treated for one year with a pyrimethamine and sulfamides dual regimen, and no ocular lesions or neurodevelopmental deficits were observed in clinical and ocular examinations performed every three months.

## Discussion

Our study indicated declining annual seroprevalence over a 7-year period, except in the youngest age group, but stable incidence of maternal and congenital *Toxoplasma* infections. Moreover, we observed that increasing age of pregnancy, multiparity, living in urban areas and region of origin were associated with seropositivity. These findings are based on a large sample from France’s second-largest public hospital system, which admits patients from a wide range of social backgrounds, living in both urban and rural areas. Another difference with national perinatal surveys was the use of a standardised set of serological tests, ensuring consistency and reliability.

The Lyon area, like other regions in the colder eastern part of France, is associated with lower seroprevalence than the national average estimates [10, 18]. In the absence of contradictory data, it can, however, be assumed that the observed decline in seroprevalence is likely to affect regions of higher or lower prevalence in Europe and the United States, where less virulent *T. gondii* Type-2 strains are also common [24]. The observed decrease in seroprevalence could be attributed to changes in lifestyles, food preparation and decontamination practices, as well as a reduction in the prevalence of *Toxoplasma* in farm animals, which can be attributed to improved farm management and enhanced biosecurity measures [17, 21]. However, the evolution of seroprevalence in farm animals remains unclear in France, as the available studies are limited to cross-sectional studies published in sheep meat [12], beef [5] and pork [8]. Another significant factor contributing to this decrease could be the declining trend in consumption of beef and sheep meat in France over the past few years [20]. Although the precise effect of cats on this decline remains uncertain, increasing urbanisation and the increased presence of urban domestic cats seem to have played a role in the decline, despite the 6% annual rate of increase of the cat population in France [11]. Unfortunately, it was not possible to estimate the risk factors to which patients had been exposed since childhood, nor to determine their educational and socio-economic level, and thus to explain the higher prevalence among patients living in urban areas, which is often reported in the literature [24].

This decline in seroprevalence has several important implications, including the diagnostic performance of serological tests in a context of lower pre-test probability and the calculation of sample sizes for prevalence studies. These consequences must be considered in the context of the serological prenatal screening, which is practiced worldwide, either through individual initiatives, or in large-scale programmes implemented in several countries. Even though, according to a recent medico-economic assessment, testing costs do not carry the highest burden compared to incidence of maternal infection which ranks first [19, 24], the search for less expensive tests is increasingly important as the number of retested patients grows. Options include using rapid immunochromatography tests [27], detecting total Ig in first line [2], or targeting at-risk populations. Our results call for regular and standardised monitoring of seroprevalence which could also be used to estimate the incidence of infection in a population [1].

In our study, the incidence of maternal infections was measured using routine screening data, which is an advantage over the available estimates, which were based on the annual number of reported congenital *Toxoplasma* cases [22], or on modelling [16]. Annual incidence estimates were overall stable over the study period. Interestingly, there was an increasing trend in the incidence of gestational infections during the SARS-CoV-2 pandemic, but which did not reach significance, unlike what was reported in an Italian study, as a possible consequence of limited access to information regarding preventive measures for toxoplasmosis and greater exposure to cats and gardening [23]. This highlights that susceptible pregnant women remain exposed to *Toxoplasma* infections, potentially even more so during pregnancy [3]. Analysis of the Lyon cohort indicated that monthly screening has reduced the risk of maternal-foetal transmission [26] and has improved the 3-year [25] and long-term outcome of infected patients [14]. However, the incidence of congenital infection has remained stable [25], and severe cases still occur, as evidenced in our limited series by the occurrence of foetal death and the detection of hyperechoic intra-cranial lesions associated with an increased risk of ocular damage [14].

## Conclusions

Our findings confirm that protecting at-risk pregnant women is a growing challenge that requires updating knowledge of local risk factors and promoting healthy behaviours. Healthcare professionals must be properly informed of all sources of infection and their prevention. Screening programmes, given their access to large populations, are best placed to assess the impact of these preventive efforts [7], provided that the data are collected with the necessary precision. Our findings also indicate the need for regular monitoring of epidemiological parameters related to maternal and congenital *Toxoplasma* infections and their prevention, using standardised protocols. These protocols should be made available to the scientific community to facilitate comparisons between studies carried out over time in a specific setting or performed in the context of different settings.
